# Supplementary material for: Specific Binding of Alzheimer’s Aβ Peptides to Extracellular Vesicles
Source: Int J Mol Sci. 2024 Mar 26;25(7):3703. doi: 10.3390/ijms25073703 (PMC11011551; doi:10.3390/ijms25073703)
Supplement: Supplementary file 1 [file ijms-25-03703-s001.zip › ijms-2854279-supplementary.pdf]

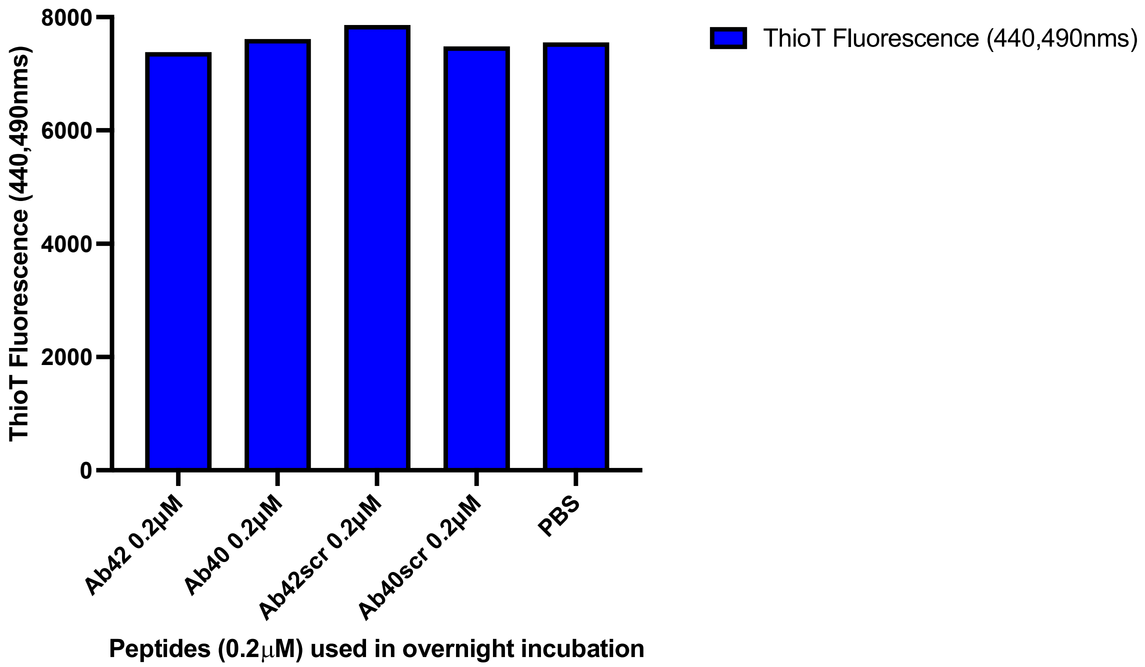

Figure S1. Starting peptides utilized for the EV-Aβ experiment have no notable β-sheet content above background. 10 μls of each of the starting Aβ peptides, utilized in the EV-Aβ-binding (Figure 2) experiments, was diluted 1:5 in PBS and read using Thio T as described in the Materials and Methods section. The starting 0.2 μM peptides were observed to have an identical ThioT fluorescence signal to a PBS buffer control, implying no β-sheet content notable above background.

Subject 23 Precipitated

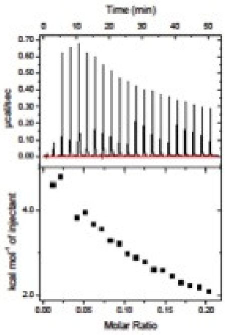

Aβ40

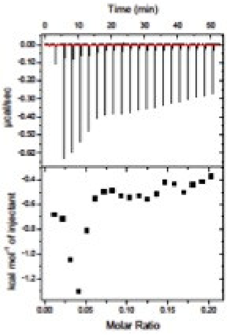

Aβ40scr

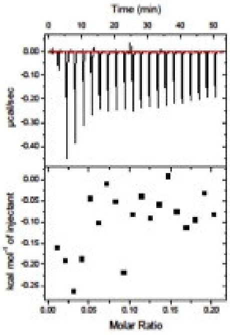

Aβ42

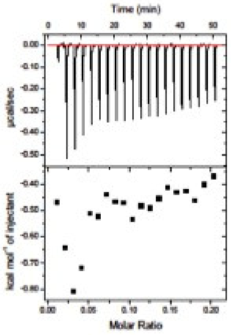

Aβ42scr

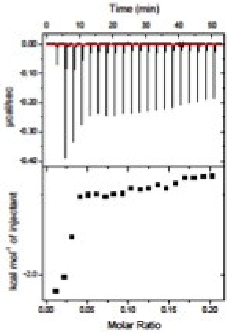

Buffer

Subject 25 Precipitated

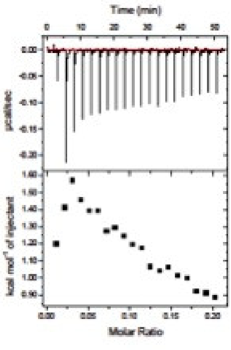

Aβ40

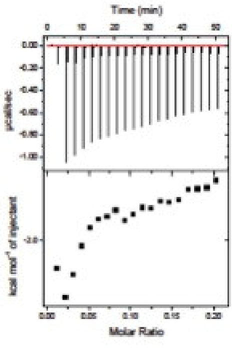

Aβ40scr

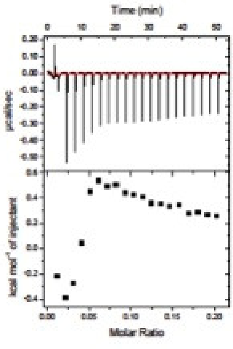

Aβ42

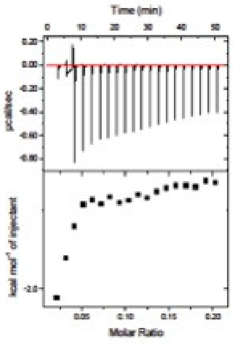

Aβ42scr

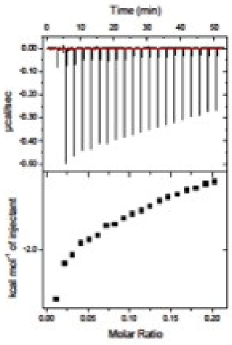

Buffer

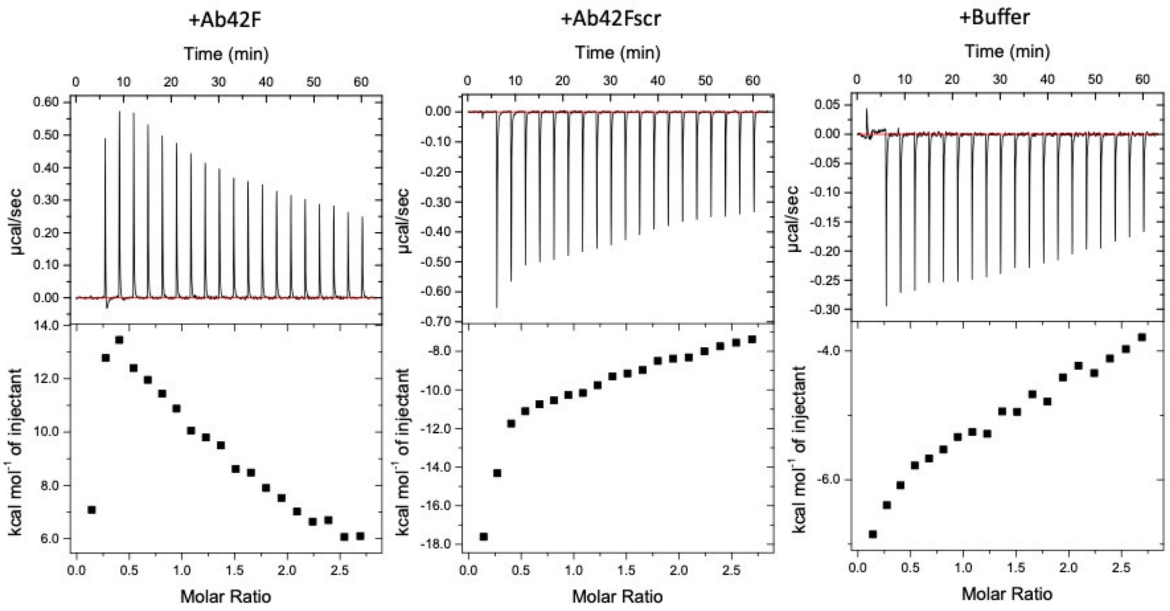

+Ab42F
+Ab42Fscr
+Buffer
Time (min)
µcal/sec
kcal mol⁻¹ of injectant
Molar Ratio

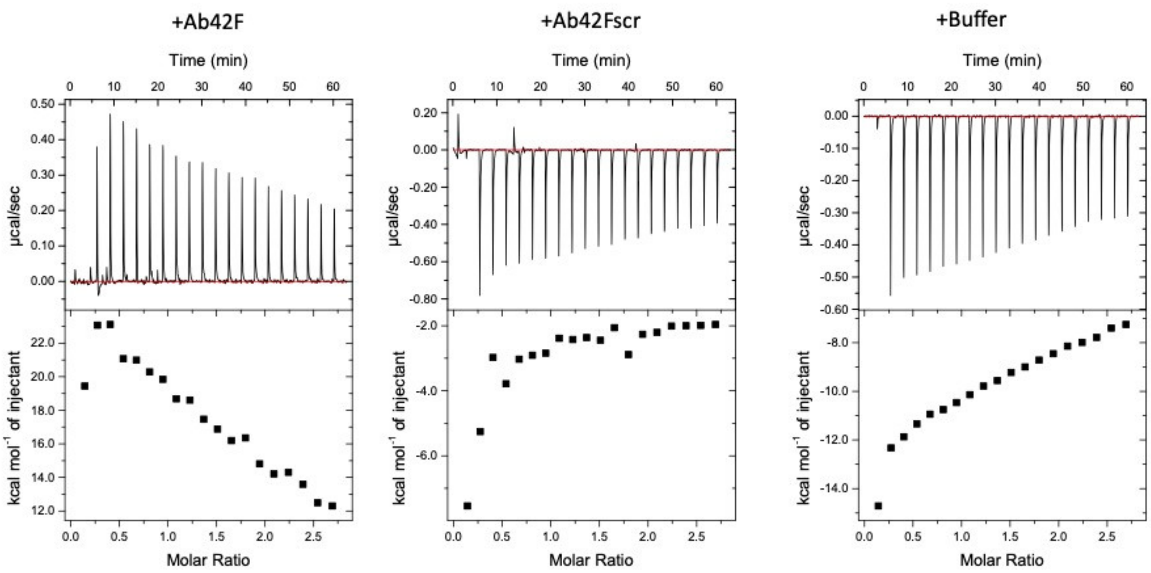
+Ab42F
+Ab42Fscr
+Buffer
Time (min)
µcal/sec
kcal mol^{-1} of injectant
Molar Ratio

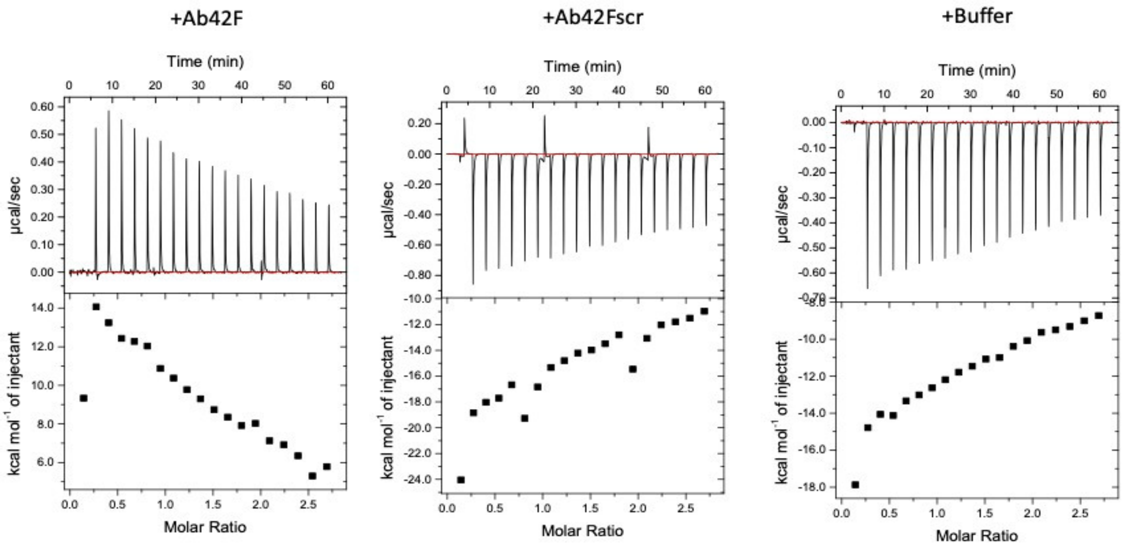

Figure S2. Repetition of the ITC. To further assess the binding of Aβ40 and Aβ42 and their scrambled versions to extracellular vesicles (EVs), we repeated the isothermal titration calorimetry (ITC) experiments an additional three times with EVs from three more control human subjects plasmas (Subject 23, 24 and 25). As previously described in Figure 3, EVs were titrated into a solution containing one of the various types and forms of Aβ peptides; Aβ42 Aβ40, Aβ42scrambled (Aβ42scr), Aβ40scrambled (Aβ40scr), Aβ42Fibrillar (Aβ42F), Aβ42Fibrillar-scrambled (Aβ42Fscr) or Buffer alone and the heat exchanges measured. The repetitions again resulted in Aβ40 and Aβ42 binding to EVs in an endothermic and saturable manner with Aβ42F continuing to show more consistent endothermic profiles than monomeric/oligomeric Aβ42..
